# Aerobic Exercise Training Attenuates Tumor Growth and Reduces Insulin Secretion in Walker 256 Tumor-Bearing Rats

**DOI:** 10.3389/fphys.2018.00465

**Published:** 2018-05-08

**Authors:** Veridiana Mota Moreira, Claudinéia Conationi da Silva Franco, Kelly Valério Prates, Rodrigo Mello Gomes, Ana Maria Praxedes de Moraes, Tatiane Aparecida Ribeiro, Isabela Peixoto Martins, Carina Previate, Audrei Pavanello, Camila Cristina Ianoni Matiusso, Douglas Lopes Almeida, Flávio Andrade Francisco, Ananda Malta, Laize Peron Tófolo, Sandra da Silva Silveira, Lucas Paulo Jacinto Saavedra, Katia Machado, Paulo Henrique Olivieri da Silva, Gabriel S. Fabrício, Kesia Palma-Rigo, Helenir Medri de Souza, Flaviane de Fátima Silva, Giuliana Regina Biazi, Taís Susane Pereira, Elaine Vieira, Rosiane Aparecida Miranda, Júlio Cezar de Oliveira, Luiz Delmar da Costa Lima, Wilson Rinaldi, Maria Ida Ravanelli, Paulo Cezar de Freitas Mathias

**Affiliations:** ^1^Laboratory of Secretion Cell Biology, Department of Biotechnology, Genetics and Cell Biology, State University of Maringá, Maringá, Brazil; ^2^Department of Physical Education, State University of Maringá, Maringá, Brazil; ^3^Department of Physical Education, Ingá University Center, UNINGÁ, Maringá, Brazil; ^4^Department of Physiological Sciences, Federal University of Goiás, Goiânia, Brazil; ^5^Department of Physical Education, Biomedical Sciences Faculty of Cacoal, Cacoal, Brazil; ^6^Department of Physiology, State University of Londrina, Londrina, Brazil; ^7^Laboratory of Microorganisms Genetics and Mutagenesis, Department of Biotechnology, Genetics and Cell Biology, State University of Maringá, Maringá, Brazil; ^8^Post-Graduate Program of Physical Education, Catholic University of Brasília, Águas Claras, Brazil; ^9^Laboratory of Endocrine Physiology, Department of Physiological Sciences, Roberto Alcântara Gomes Biology Institute, State University of Rio de Janeiro, Rio de Janeiro, Brazil; ^10^Institute of Health Sciences, Federal University of Mato Grosso, Sinop, Brazil; ^11^Superior School of Physical Education and Physical Therapy of Goiás State, State University of Goiás, Goiânia, Brazil; ^12^Department of Physiology, State University of Maringá, Maringá, Brazil

**Keywords:** Walker 256 tumor cells, aerobic exercise training, metabolism, insulin secretion, pancreatic islets

## Abstract

Aerobic exercise training can improve insulin sensitivity in many tissues; however, the relationship among exercise, insulin, and cancer cell growth is unclear. We tested the hypothesis that aerobic exercise training begun during adolescence can attenuate Walker 256 tumor growth in adult rats and alter insulin secretion. Thirty-day-old male Wistar rats engaged in treadmill running for 8 weeks, 3 days/week, 44 min/day, at 55–65% VO_2max_ until they were 90 days old (TC, Trained Control). An equivalently aged group was kept inactive during the same period (SC, Sedentary Control). Then, half the animals of the SC and TC groups were reserved as the control condition and the other half were inoculated with Walker 256 cancer cells, yielding two additional groups (Sedentary Walker and Trained Walker). Zero mortalities were observed in tumor-bearing rats. Body weight (BW), food intake, plasma glucose, insulin levels, and peripheral insulin sensitivity were analyzed before and after tumor cell inoculation. We also evaluated tumor growth, metastasis and cachexia. Isolated pancreatic islets secretory activity was analyzed. In addition, we evaluated mechanic sensibility. Our results showed improved physical performance according to the final workload and VO_2max_ and reduced BW in trained rats at the end of the running protocol. Chronic adaptation to the aerobic exercise training decreased tumor weight, cachexia and metastasis and were associated with low glucose and insulin levels and high insulin sensitivity before and after tumor cell inoculation. Aerobic exercise started at young age also reduced pancreatic islet insulin content and insulin secretion in response to a glucose stimulus, without impairing islet morphology in trained rats. Walker 256 tumor-bearing sedentary rats also presented reduced pancreatic islet insulin content, without changing insulin secretion through isolated pancreatic islets. The mechanical sensitivity test indicated that aerobic exercise training did not cause injury or trigger inflammatory processes prior to tumor cell inoculation. Taken together, the current study suggests that aerobic exercise training applied during adolescence may mitigate tumor growth and related disorders in Walker 256 tumor-bearing adult rats. Improved insulin sensibility, lower glucose and insulin levels and/or reduced insulin secretion stimulated by glucose may be implicated in this tumor attenuation.

## Introduction

Cancer is one of the great challenges of science and health global, due to the epidemiological profile, suffering, and death that this disease has presented (Siegel et al., 2016; Torre et al., 2016; WHO, 2018b). Each year, eight million people die from cancer, making it a leading cause of death worldwide; until 2035, it is predicted that nearly fifteen million deaths will be related to cancer (WHO, 2018b). According to current evidence, it is known that 30% and 50% of these deaths could be prevented by modifying or avoiding key risk factors, including avoiding tobacco products, reducing alcohol consumption, maintaining a healthy body weight (BW), exercising regularly and addressing infection-related risk factors (Campbell and McTiernan, 2007; Vermaete et al., 2013; WHO, 2018a).

Experimental tumors in rodents are considered one of the main tools of preclinical screening before clinical testing in humans (de Jong and Maina, 2010), providing results that will be critical to informing hypothesis-driven clinical trials and ensuring the optimal safety and efficacy of different protocols (Betof et al., 2013). In this regard, exercise has been proposed as a no pharmacological therapy to prevent and alleviate cancer tumors and the adverse consequences of cancer (Newton and Galvão, 2008; Almeida et al., 2009; Segal et al., 2017). Early in life, in human, exercise is associated on adult health outcomes including cancer control and all-cause mortality (Fuemmeler et al., 2009; Betof et al., 2013; Nechuta et al., 2015).

Breast cancer Walker 256 tumor-bearing rats are an animal model frequently used by different research lines to study tumor growth, cachexia, and metastasis (Earle, 1935; Guaitani et al., 1982; Moraes et al., 2000). These cells are easily cultivated and transplanted. To our knowledge, no study has been conducted to examine the protective effects of aerobic exercise performed by adolescent male rats, in which the Walker 256 tumor cells are inoculated after the last session of the training, when reaching adulthood. Effects of the same type of exercise in the pancreatic morpho-physiology of young animals and the maintenance or not of these, in adult sedentary life, especially related to cancer progression, has not been reported.

Although the mechanisms underlying on cancer prevention and progression are still not fully understood, there is evidence that chronic moderate exercise induce several desirable metabolic alterations, such as reducing fat cell volume (Nechuta et al., 2015; Pistor et al., 2015), improving tissue insulin sensitivity and reducing fasting hyperinsulinemia (Westerlind et al., 2003; Gomes et al., 2013; Keshel and Coker, 2015) and increase pancreatic beta-cell function and mass (Calegari et al., 2012; Gomes et al., 2013; Paula et al., 2018; Veloso et al., 2018). This information is very important, because during tumor growth and cell proliferation, weak pancreatic insulin secretion is present (Fernandes et al., 1990; Rose and Vona-Davis, 2012).

The aim of this study was to investigate the effects of aerobic exercise training starting at adolescence, on Walker 256 tumor growth and insulin secretion in adult rats.

## Materials and Methods

### Animals and Experimental Groups

All experiments were performed in accordance with the standards of the United Kingdom Coordinating Committee of Cancer Research (Workman et al., 2010) and approved by the Ethics Committee of the State University of Maringá (protocol number 2585010516). Male Wistar rats were obtained at 21 days old and were kept in appropriate cages (five rats per cage) under controlled temperature conditions (22 ± 2°C), a light/dark cycle of 12 h (7:00 am–7:00 pm) and *ad libitum* access to water and a standard diet (Nuvital^®^, Curitiba, Brazil).

**FIGURE 1:**
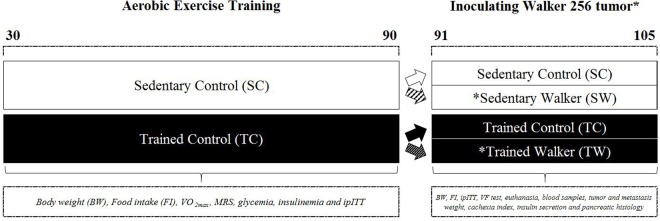
Schematic overview of the protocols. The aerobic exercise training was performed at beginning of adolescence. Male rats with ages of 30 days were submitted to a running treadmill protocol until they reached 90 days of age (Trained Control, TC). An equivalent group was kept inactive during the same period (Sedentary Control, SC). After this, half of the animals belonging to the SC and TC remained in the control condition and the other half of the animals were inoculated with Walker 256 cancer cells, yielding two more groups. On the 15th day of inoculation, four groups of both ages were euthanatized to conduct subsequent analyzes.

At 30 days old, at beginning of adolescence (Evans, 1986), the animals were randomized into two groups: Sedentary Control (SC) and Trained Control (TC) (**Figure 1**). TC rats performed an aerobic treadmill running protocol (Panlab, Harvard Apparatus^®^, LE8700C 76-0553, Cornellà, Barcelona, Spain) until reaching 90 days old or adulthood (Evans, 1986; Jackson et al., 2017) (*First phase of this study*). Then, half of the animals from both groups remained under control conditions (PBS inoculation) and the other half were inoculated with Walker 256 cancer cells (on day 91), giving rise to two additional groups: Sedentary Walker (SW) and Trained Walker (TW) (*Second phase of this study*) rats (**Figure 1**).

On the 15th day (105 days old) after inoculation with cancer cells (SW and TW groups) or PBS (SC and TC groups), the animals of the four groups were euthanized for collection of blood and removal of the pancreas, tumor and metastases for subsequent analysis.

### Physical Fitness Test

All rats performed a physical fitness test to determine their individual maximal oxygen uptake (VO_2max_) and maximal running speed (MRS). The test utilized a gas analyzer coupled to a treadmill for rodents (Panlab, Harvard Apparatus^®^, LE405 76-0195 O_2_/CO_2,_ Cornellà, Barcelona, Spain). The test began with a warm up (5 min, 10 cm/s, 0° of inclination), after which the velocity was increased by 9 cm/s every 3 min until exhaustion of the animal to obtain VO_2max_ and MRS, using Metabolism software, version 2.2.02. The decision to use 3 min at each stage was previously described by Wisløff et al. (2001), who reported that oxygen consumption stabilized after approximately 3 min at each stage of exercise after a change in workload (Wisløff et al., 2001). At the end of the treadmill line, a stainless steel grid emitted electrical stimuli (0.2 mA in < 1 s) to keep the animal in motion, as previously reported (Jones, 2007). The animal’s inability to maintain the pace was considered to be a sign of exhaustion (Rodrigues et al., 2007). A physical fitness test was performed before (initial: 30 days old) and after (final: 90 days old) the aerobic exercise training period. Incremental tests were performed every 15 days to adjust the training load.

### Training Protocol

The protocol of dynamic aerobic exercise training was adapted from Caponi et al. (2013). Previous adaptation was performed in five sessions with durations of 10, 12, 14, 16, and 18 min and an intensity of 16 cm/s. Two days of rest were given prior to applying the physical fitness test. The animals ran at low-moderate intensity (50–65% of MRS obtained during the physical fitness test) for 44 min a day, from 8 to 11 a.m., 3 days a week for 8 weeks, with a gradual increase in velocity every 15 days.

### Biometric and Metabolic Parameters

Body weight and food intake were measured weekly during the first phase of the study and on alternate days during the second phase of the study. Food intake was evaluated by determining the difference between the amount of food remaining and total amount that was initially placed in the cage divided by the number of animals in the box and the number of days. Data are presented as absolute values (g, for BW) and relative values (g per 100 g of BW, for food intake). In addition, the area under the curve (AUC), for both parameters, was calculated.

In order to characterize the first experimental phase of this study, in a prudent and rational manner, and to keep the sample without euthanasia and at the same time reduce the request of animals (Russell et al., 1959), blood was collected through a small distal cut in the tail for immediate collection of blood glucose and insulin measurement. The glycemic values were obtained with the help of the glucometer Abbott Free Style Optimum H (Abbott Park, IL, United States) which uses specific and disposable reactive strips. Insulin was measured by radioimmunoassay (RIA) (Scott et al., 1981) with a gamma counter through a gamma particle emission counter (Wizard2 Automatic Gamma Counter, TM-2470, PerkinElmer^®^, Shelton, CT, United States), using standard rat insulin, anti-rat insulin (Sigma-Aldrich^®^, St Louis, MO, United States) and recombinant human insulin ([^125^I]-Insulin (h)] (PerkinElmer^®^, Shelton, CT, United States).

At the end of the second experimental phase or after 15 days of tumor cell inoculation and 12 h of fasting, animals from all groups were weighed and decapitated, and blood samples were collected and centrifuged (10,000 rpm for 5 min) to obtain plasma for further biochemical analysis. The plasma was used to measure glucose by the enzymatic method using a commercial colorimetric kit (Gold Analisa^®^, Belo Horizonte, Brazil) and quantified by spectrophotometry (BIO200FL, Bio Plus^®^, São Paulo, Brazil). The analysis of insulinemia proceeded as previously described.

### Intraperitoneal Insulin Tolerance Test (ipITT)

The Intraperitoneal Insulin Tolerance Test (ipITT) was performed at the end of the eighth week of exercise training and after 13 days of tumor cell inoculation. Food was withdrawn 6 h before the test, and free access to water was allowed. The rats received an intraperitoneal injection of human recombinant insulin (1 U/kg of BW; Eli Lilly^®^, São Paulo, Brazil). Blood samples were obtained through tail venesection, and glucose was measured using a glucometer (Abbott Free Style Optimum H, Abbott Park, IL, United States) at 0 (3:00 p.m.; prior to insulin injection), 15, 30, 45, and 60 min after injection. Thereafter, the constant rate for glucose disappearance (K_itt_) was calculated, as previously described (Lundbaek, 1962). The plasma glucose t_1/2_ was calculated from the slope of the least squares analysis of plasma glucose concentrations during the linear decay phase. Anesthesia was not used during the ipITT test (Calegari et al., 2011).

### Inoculation of Walker 256 Tumor Cells

Walker 256 tumor cells, maintained intraperitoneally in rats, were collected, centrifuged and resuspended in phosphate buffered saline [PBS (mmol l^-1^): 16.5 phosphate; 137 NaCl and 2.7 KCl, pH 7.4], and cell viability was evaluated using the trypan blue exclusion method. At 91 days old, half of the sedentary and trained rats were inoculated with 8.0 × 10^7^ viable tumor cells/animal in the right rear flank (Franco et al., 2014). In the control groups, PBS was inoculated at the same rear flank site. Inoculation with the Walker 256 tumor cells was performed 24 h after last training session (**Figure 1**).

### Tumor Growth, Cachexia, Carcass Weight, and Metastasis Evaluation

After 15 days of tumor cell inoculation, each tumor mass was carefully dissected and weighed, and total carcass weight (total body weight minus the tumor weight) was measured. The percentage of body weight loss (BWL) or cachexia index was determined in order to fix a pattern of general wasting, considering initial and final BW of the rats with tumor, tumor weight, and body weight gain in control groups, using the following equation (Guarnier et al., 2010; Fracaro et al., 2016):

$$\begin{array}{c} \left[Initial\;body\;weight\;(g)\;-\;final\;body\;weight\;(g\right)\\ \underline{+\;tumor\;mass\;(g)\;+\;body\;weight\;gain\;(g)]\;\times \;100}\\ \left[Initial\;body\;weight\;(g)\;+\;body\;weight\;gain\;(g)\right] \end{array}$$

The rats were considered to be cachectic when BWL was higher than 10% (Trombini et al., 2015). Metastases from the retroperitoneal region were also removed. Tumor mass values were expressed relative to 100 g of BW.

### Pancreatic Islet Isolation

At 105 days old, pancreatic islets were isolated from rats of all groups using the collagenase method and washed with Hanks’s solution, as previously described (Gravena et al., 2002). For this, rats were decapitated, and their abdominal walls were cut and opened. Then, the common bile duct was injected with 8 ml of Hanks buffered saline solution [HBSS (in mmol l^-1^): 136.9 NaCl; 5.4 KCl; 0.81 MgSO_4_7H_2_O; 0.34 Na_2_HPO_4_; 0.44 KH_2_PO_4_; 1.26 CaCl_2_2H_2_O; 4.16 NaHCO_3_; 0.06 glucose and 15 bovine serum albumin (BSA)], containing 0.1% of collagenase type XI, 5% of BSA and 0.6% of HEPES [*N*-(2-hydroxyethyl-piperazine)-*N*’-(2-ethanesulphonic acid)] (Sigma-Aldrich^®^, St. Louis, MO, United States). The HBSS was prepared in a gas mixed (95% of O_2_ and 5% of CO_2_) per 10 min and after adjusted to pH 7.4. The pancreas was swollen with the collagenase solution and then quickly excised and incubated in a glass beaker for 17–18 min at 37°C. The suspension was then discarded and washed with HBSS in three continuous washings. The islets were collected with the aid of a stereomicroscope.

### Insulin Secretion Stimulation and Total Content of Insulin

To adapt the isolated islets to a baseline glucose concentration (5.6 mmol l^-1^), groups of four islets were pre-incubated for 60 min in 1 ml of normal Krebs-Ringer solution: [HBSS (mmol l^-1^): 115 NaCl; 1.6 KCl; 1 MgCl_6_H_2_O; 24 NaHCO_3_; 1 CaCl_2_2H_2_O; 5.6 glucose and 15 BSA], in O_2_, 95% + CO_2_, 5% mixed/10 min, pH 7.4. To study the insulinotropic effect of glucose, after pre-incubation, the islets were incubated for an additional 60 min in Krebs-Ringer solution containing different concentrations of glucose [5.6; 8.3; 11.1 and 16.7 mmol l^-1^].

The insulin levels were measured by RIA (Scott et al., 1981). Standard human insulin, anti-rat insulin antibody (Sigma-Aldrich^®^, St. Louis, MO, United States) and recombinant human insulin labeled Iodo^125^ (PerkinElmer^®^, Shelton, CT, United States) were used. The intra- and interassay coefficients of variation were 9.8% and 12.2%, respectively, for insulin. The insulin level of the detection limit was 0.006 ng/ml, and measurements were performed in a single assay.

To evaluate the total insulin content, the islets were incubated in 1 ml acid-ETOH buffer (1.5 ml HCl in 100 ml 70% ETOH) for 24 h at 4°C. Then, the samples were homogenized and centrifuged at 3,000 rpm for 15 min at 4°C, and the supernatant was collected and diluted in Krebs-Ringer solution (1:800; v/v) and then stored at -20°C before measuring the total insulin content by RIA assay (Scott et al., 1981), as described above.

### Histological Analyses of the Endocrine Pancreas

Pancreas samples from 105-day-old rats were fixed in 10% buffered formalin, dehydrated, embedded in histological paraffin and sectioned (5 μm) in non-serial cuts. The tissue sections were deparaffinized, rehydrated and stained with hematoxylin and eosin (HE) or immunostained with anti-insulin antibody, as described below. Sections were blocked against endogenous peroxidase, washed in phosphate buffer 0.01 mol/L (PBS, pH 7.4) and incubated with 10% non-immune goat serum blocking solution (Histostain-Plus^®^, Invitrogen, Carlsbad, CA, United States) for 10 min. Then, the sections were incubated with monoclonal primary antibody against insulin at a dilution of 1:500 (Sigma^®^, St. Louis, MO, United States) for 60 min. After washing (0.01 mol/l PBS), the sections were incubated with a biotinylated secondary antibody (Histostain-Plus^®^) for 10 min, washed and incubated with diaminobenzidine chromogenic solution (Histostain-Plus^®^) for 15 min, and finally washed and counterstained with hematoxylin.

The morphometric analyses were performed using digital images (TIFF 24-bit color, 2560 × 1920 pixels) obtained using light microscopy (Olympus BX41, Tokyo, Japan) and a camera (QColor 3, Olympus). Analyses were performed using Image-Pro Plus 4.5 software (Media Cybernetics, Silver Spring, MD, United States). To determine pancreatic islets number digital images (×20 magnification) from six different fields from each animal (*n* = 4 animals/group) were used. The islets number (number of islets/mm^2^) was obtained by measuring of the number of pancreatic islets by the total area of randomly selected images. Analyses of the islet area and β-cell mass were performed using 40 digital images (×400 magnification) from each animal (*n* = 4 animals/group). Measurements of islet area were performed by Image-Pro Plus using specific area measurement tools. The relative beta-cell mass per pancreas area, was determined by point counting morphometry on each pancreas section immunostained for insulin according previous descriptions (Xu et al., 1999; Rafacho et al., 2009; Malta et al., 2016; Gomes et al., 2017).

### Nociceptive Test

The nociceptive test (von Frey) was performed for 12 days, on alternate days after inoculation of tumor cells or PBS, between 9 and 11 a.m. The von Frey test is a nociceptive test in which filaments with different bucking forces are applied to the skin surface to evaluate the development of allodynia, a type of mechanic hypersensitivity, in animal models. With the aim to evaluate whether physical training at a young age can prevent hypernociception induced by tumor growth, tumor-bearing sedentary and trained rats were placed on an elevated wire grid, and the plantar surfaces of their hind paws were stimulated with a series of ascending force von Frey (VF) monofilaments (Stoelting Co., Wood Dale, IL, United States).

To perform the test, the animals were first habituated to the experimental environment (room and apparatus) for a period of at least 30 min. Mechanical allodynia was assessed by measuring the paw withdrawal threshold taken as the lowest force that evoked a brisk withdrawal response to one of five repetitive stimuli. Briefly, a logarithmic series of 10 calibrated monofilaments (VF hairs) was applied to the right hind paws to determine the stimulus intensity threshold stiffness required to elicit a paw withdrawal response. The log stiffness of the hairs was determined by log10 mg and ranged from 0.903 (8 mg or 0.008 g) to 3.0 (1000 mg or 1 g). The mechanical threshold was represented as previously described (Tal and Bennett, 1994; Hains et al., 2010). An investigator who was blinded to the group allocation performed all behavior experiments. Data are presented in the log scale from baseline (BL) and as the AUC.

### Statistical Analysis

Student’s *t*-test was used to compare VO_2max,_ MRS and metabolic parameters from SC and TC groups, along the first phase and to compare tumor weight, cachexia, and metastasis of the inoculated groups in the second phase of the study. Two-way ANOVA considering tumor cell inoculation and aerobic exercise training as the factors was employed to compare the general data from SC, TC, SW, and TW groups in the second phase of the study. Tukey’s *post hoc* test was employed to compare and show the means between groups. Repeated measures ANOVA with Sidak’s multiple comparisons test was performed to analyze the evolution of BW, food intake, and blood glucose levels measured at different time points during ipITT in both experimental phases. Data are presented as the mean ± SEM, and statistical significance was set at *p* < 0.05. Analysis and construction of graphs were performed with GraphPad Prism software (Version 6.01, La Jolla, CA, United States).

## Results

### VO_2max_ and MRS

**Table 1** shows that the rats at the end of aerobic exercise training demonstrated significant increases in VO_2max_ (*p* = 0.0007) and MRS (*p* = 0.0002) compared to the sedentary rats. No difference was observed in the initial values, indicating homogeneity of the experimental groups before starting the training protocol.

**Table 1:** VO_2max_ and MRS.

Parameters	Initial			Final		
	SC	TC	*P*-value	SC	TC	*P*-value
VO_2max_ (ml/min/kg^∧^0.75)	21.40 ± 0.35	21.10 ± 0.64	NS	15.66 ± 0.84	23.48 ± 0.52	<0.0001
MRS (cm/s)	43.35 ± 2.15	44.41 ± 1.93	NS	33.5 ± 2.657	56.69 ± 3.31	<0.0001

*The data represent the mean ± SEM of the initial and final VO_2max_ and the MRS of 14–17 rats based on Student’s oxt-test. SC, Sedentary Control; TC, Trained Control.*

### Tumor Weight, Cachexia, Carcass Weight, and Metastasis

**FIGURE 2:**
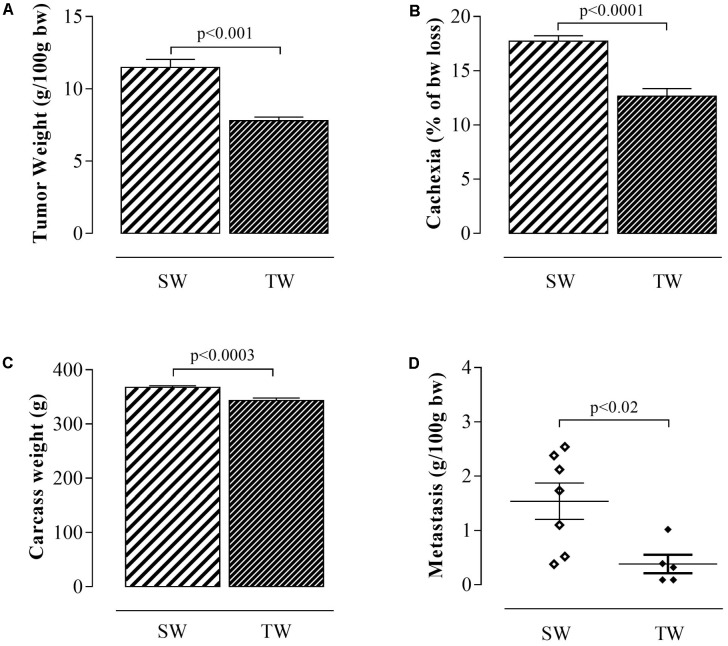
Tumor weight, cachexia, carcass weight and metastasis in rats inoculated with Walker 256 tumor cells. The values are presented as the mean ± SEM of the relative tumor weight **(A)**, cachexia **(B)**, carcass weight **(C)**, and metastasis **(D)**. The data were obtained from 5 to 9 rats. Student’s *t*-test was used for group comparisons. SW, Sedentary Walker; TW, Trained Walker.

Walker 256 tumor-bearing trained rats showed significant reductions in tumor growth (*p* < 0.001; **Figure 2A**), cachexia (*p* < 0.0001; **Figure 2B**), carcass weight (*p* < 0.0003; **Figure 2C**), and metastasis (*p* < 0.02; **Figure 2D**) compared to the sedentary rats.

### Biometric and Metabolic Parameters

Significant differences were detected in BW in the trained rats, with a reduced AUC before the tumor cell inoculation compared to the sedentary rats (*p* < 0.002, **Table 2**). No difference was observed in the AUC of food intake of the trained rats (data not shown).

**Table 2:** Biometric and metabolic parameters.

Parameters	Before tumor cells inoculation			After tumor cells inoculation				*P*-value		
	SC	TC	*P*-value	SC	SW	TC	TW	1	2	3
AUC BW (g)	2998 ± 28	2766 ± 50	0.0002	2739 ± 43	2800 ± 12	2678 ± 68	2608 ± 3*l*^Ω^	0.005	0.923	0.128
Glycemia (mg/dl)	81.17 ± 1.31	72.50 ± 0.98	<0.0001	89.62 ± 1.77	76.71 ± 1.63^ψ^	84.14 ± 2.60	73.86 ± 1.06^ζ^	0.03	<0.0001	0.474
Insulinemia (ng/ml)	0.18 ± 0.02	0.12 ± 0.02	<0.01	0.24 ± 0.02	0.10 ± 0.02^ψ^	0.16 ± 0.01^ψ^	0.08 ± 0.02^ζ^	0.001	<0.0001	0.060
K_itt_ (% min)	1.57 ± 0.05	1.74 ± 0.05	<0.01	1.32 ± 0.13	1.48 ± 0.1	1.35 ± 0.1	1.9 ± 0.09^ζ^	0.05	0.005	0.081

*The data represent the mean ± SEM obtained from 14 to 18 rats before tumor cell inoculation and 5–9 rats after inoculation. Student’s oxt-test was used to compare the groups before tumor cell inoculation. Two-way ANOVA and Tukey’s post hoc test were used for group comparisons after tumor cell inoculation (1 = effect of aerobic exercise training, 2 = effect of tumor cell inoculation, 3 = interaction between training and inoculation). ^Ω^Compared with SW, ^ψ^compared with SC, ^ζ^compared with TC. SC, Sedentary Control; TC, Trained Control; SW, Sedentary Walker, TW, Trained Walker.*

**FIGURE 3:**
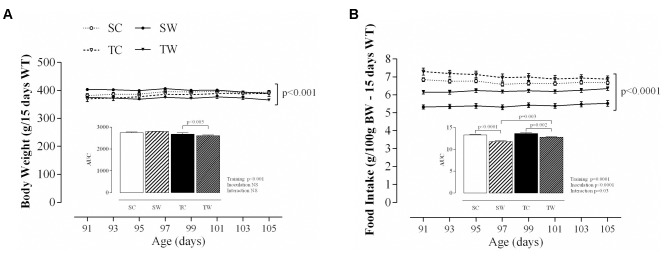
Body weight (BW) and food intake. The values are presented as the mean ± SEM of BW **(A)** and food intake **(B)** obtained from eight rats after tumor cell inoculation. The AUCs of BW and food intake are presented in each figure. Repeated measures ANOVA with Sidak’s *post hoc* test was performed to analyze the evolution of BW and food intake. SC = Sedentary Control, TC = Trained Control, SW = Sedentary Walker, TW = Trained Walker.

During 15 days of tumor growth, the rats of all groups exhibited different BW (*p* < 0.001; **Figure 3A**) and food intake (*p* < 0.001; **Figure 3B**). In addition, tumor-bearing trained rats also showed a significant decrease in the AUC of BW (*p* < 0.005; **Figure 3A**) and food intake (*p* = 0.002; **Figure 3B**) compared to trained rats without tumors. Tumor-bearing sedentary rats showed a significant decrease in the AUC of food intake (*p* = 0.003; **Figure 3B**) compared to the SC and tumor-bearing trained rats.

Aerobic training significantly reduced fasting glucose before tumor cell inoculation (*p* < 0.0001; **Table 2**) and insulin levels (*p* < 0.01; **Table 2**) in exercised rats compared to sedentary rats. Tumor cell inoculation provoked a significant glucose reduction in tumor-bearing sedentary rats compared to sedentary rats (*p* < 0.002, **Table 2**), as well as an effect related to aerobic training in tumor-bearing exercised rats compared to equivalent control rats (*p* < 0.003; **Table 2**).

Aerobic exercise training and tumor cell inoculation affected fasting insulin levels in different manners. Even after ceasing the aerobic exercise protocol, the trained rats without tumors maintained low insulin levels (*p* < 0.02; **Table 2**). Tumor-bearing trained rats showed a reduction in insulin levels compared to their counterparts without tumors (*p* < 0.01; **Table 2**). Similarly, tumor-bearing sedentary rats showed a reduction in insulin levels compared to sedentary rats without tumors (*p* < 0.0001; **Table 2**).

### Intraperitoneal Insulin Tolerance Test

**FIGURE 4:**
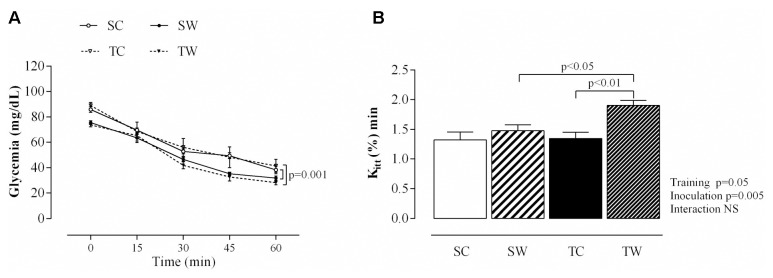
Aerobic exercise training improves insulin sensitivity. The values are presented as the mean ± SEM of blood glucose levels during the intraperitoneal insulin tolerance test (ipITT) **(A)** and blood glucose removal rate (K_itt_) **(B)** during intraperitoneal ipITT, obtained from six rats after tumor cell inoculation. Repeated measures ANOVA with Sidak’s *post hoc* test was performed to analyze glucose concentrations after insulin injection. SC, Sedentary Control; TC, Trained Control; SW, Sedentary Walker; TW, Trained Walker.

After insulin injection, glucose concentrations were decreased in the tumor-bearing sedentary rats at 15, 30, 45, and 60 min compared to sedentary rats (*p* = 0.001; **Figure 4**). Tumor-bearing trained rats also showed reduced blood glucose after insulin injection compared to counterparts without tumors at the same collection times (*p* = 0.001; **Figure 4**).

In addition, a significant increase in blood glucose disappearance rate during ipITT was detected in trained rats before tumor inoculation compared to sedentary rats (*p* < 0.01; **Table 2**). This result did not remain 13 days after the last training session, but there was an increased K_itt_ in tumor-bearing trained rats compared to their counterparts without tumors (*p* < 0.01, **Table 2** and **Figure 4**) and compared to Walker 256 tumor-bearing sedentary rats (*p* < 0.05, **Table 2** and **Figure 4**).

### Insulin Secretion and Total Insulin Content

**FIGURE 5:**
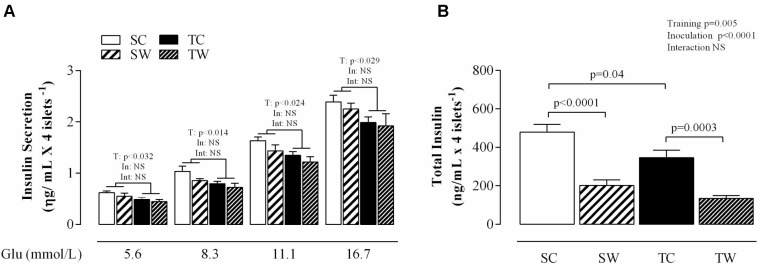
Glucose-induced insulin release and total insulin content from pancreatic islets of isolated rats. The values are presented as the mean ± SEM. of insulin secretion obtained from 5 to 7 rats **(A)** and total insulin content **(B)**. Two-way ANOVA with Tukey’s *post hoc* test was used for group comparisons (T = effect of aerobic exercise training, In = effect of tumor cell inoculation, Int = interaction between training and inoculation). SC, Sedentary Control; TC, Trained Control; SW, Sedentary Walker; TW, Trained Walker; NS, not significant.

The aerobic training conferred a significant decrease in insulin secretion from islets isolated in response to all glucose concentrations in exercised rats compared to sedentary rats (5.6 mmol l^-1^: *p* < 0.014; 11.1 mmol l^-1^: *p* < 0.024 and 16.7 mmol l^-1^: *p* < 0.029; **Figure 5A**). Tumor cell inoculation did not change the glucose-induced insulin secretion from islets isolated from Walker 256 tumor-bearing rats compared to rats without tumors (**Figure 5A**).

Training and tumor cell inoculation showed distinct effects on total insulin content. Aerobic exercise training reduced total insulin content (*p* < 0.04; **Figure 5B**) compared to the sedentary condition. Similarly, Walker 256 tumor-bearing trained rats showed reductions in total insulin content compared to similar non-inoculated rats (*p* = 0.0003; **Figure 5B**). Walker 256 tumor-bearing sedentary rats demonstrated reductions in total insulin content compared to similar non-inoculated rats (*p* < 0.0001, **Figure 5B**). No differences were observed between the sedentary and trained tumor-bearing rats.

### Histological Analyses of the Endocrine Pancreas

**FIGURE 6:**
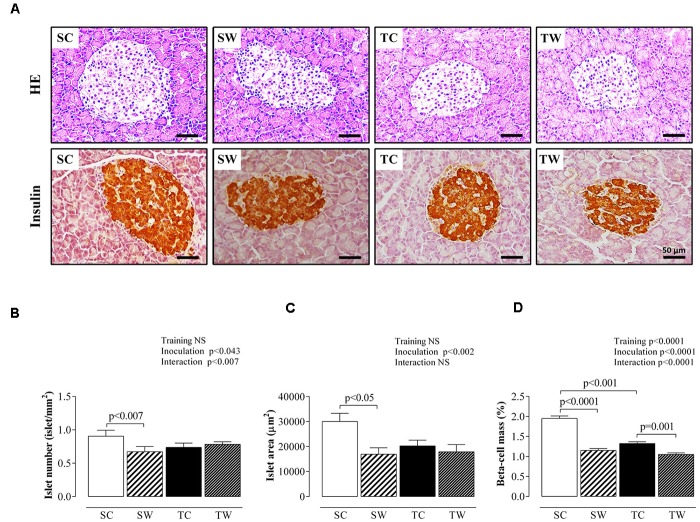
Endocrine pancreas morphology. **(A)** Representative photomicrography (×400 magnification, scale bars = 50 μm) shows pancreatic sections stained with hematoxylin and eosin (HE) and pancreatic sections immunostained with anti-insulin antibody. Quantitative analyses of islet number, islet area, and beta-cell mass are shown in **(B–D)**, respectively. Data are presented as the mean ± SEM and were obtained from four rats in each experimental group. A total of 160 islets were analyzed per group. Two-way ANOVA and Tukey’s *post hoc* test were used for group comparisons (Training = the effect of aerobic exercise training, Inoculation = the effect of tumor cell inoculation, Interaction = interaction between training and inoculation). SC, Sedentary Control; TC, Trained Control; SW, Sedentary Walker; TW, Trained Walker; NS, not significant.

**Figure 6** shows histological and morphological analyses of the endocrine pancreas. Walker 256 tumor-bearing sedentary rats demonstrated reductions in pancreatic islet number (*p* < 0.007, **Figure 6B**), islet area (*p* < 0.05, **Figure 6C**) and beta-cell mass (*p* < 0.0001, **Figure 6D**) compared to non-inoculated rats. No differences were observed in pancreatic islet numbers and islet areas of both inoculated and non-inoculated trained rats (**Figures 6B,C**, respectively).

Training and tumor cell inoculation induced similarly effects on beta-cell mass. Rats whose exercise started at adolescence and stopped the protocol for 15 days, presented a reduced beta-cell mass (*p* < 0.001; **Figure 6D**) compared to the sedentary condition. Similarly, Walker 256 tumor-bearing trained rats exhibited reduced beta-cell mass compared to non-inoculated rats (*p* = 0.001, **Figure 6D**).

### von Frey Test

**FIGURE 7:**
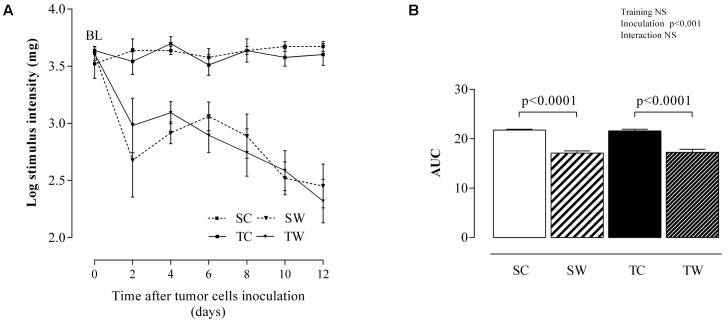
Mechanical sensitivity from day 0 to 12 post inoculation. Stimulus intensity **(A)** and AUC **(B)** during the VF test. The values are presented as the mean ± SEM of 5–7 rats. Two-way ANOVA with Tukey’s *post hoc* test was used for group comparisons (Training = effect of aerobic exercise training, Inoculation = effect of tumor cell inoculation, Interaction = interaction among training and inoculation). BL = baseline, SC = Sedentary Control, TC = Trained Control, SW = Sedentary Walker, TW = Trained Walker, NS = not significant.

The von Frey test showed that both trained and sedentary groups had similar baseline (prior to inoculation) sensitivity to mechanical stimuli, an indirect indication that the intensity of aerobic exercise training applied did not cause muscle injury or inflammation in the paw (**Figures 7A,B**). On the other hand, tumor growth caused a great hypersensitivity to the mechanical stimulus (allodynia) during the 12 days following inoculation with tumor cells, as evidenced by the intense decrease in the response threshold to the mechanical stimulus, both in the sedentary groups (*p* < 0.0001, **Figure 7B**) and in the trained groups (*p* < 0.0001, **Figure 7B**). The decrease in the response threshold can be interpreted as indicative of an inflammatory process caused by tumor growth, which was not prevented by aerobic exercise training during adolescence (**Figures 7A,B**).

## Discussion

We found that chronic exercise at moderate intensity and low frequency, beginning at 30 days old, was able to attenuate tumor growth and reduce insulin secretion in Walker 256 tumor-bearing adult (90 days old) rats. Moreover, for the first time, we demonstrated that aerobic exercise training applied during adolescence was also able to reduce metastatic foci and prevent cancer-related cachexia in Walker 256 tumor-bearing adult (90 days old) rats. In this study, we explored the glucose-insulin signaling implications on biometric, biochemical, morphological and physiological parameters through which aerobic exercise training could positively or negatively influence oncogenic pathways.

A reduction in tumor growth may be dependent on the type and intensity of exercise. Swim training at 50% of maximal workload (but not at 80%) significantly reduced the weight of Ehrlich tumors in young Swiss mice (Almeida et al., 2009). Moderate exercise training during the pre- and early pubescent period was also associated with the retardation in the mammary tumors growth in Sprague-Dawley rats (Westerlind et al., 2003). In our study, Wistar rats ran on a treadmill at low-moderate intensity (50–65% of MRS) for 44 min a day, 3 days a week for 8 weeks, with a gradual increase in velocity every 15 days. Aerobic exercise training was able to improve functional capacity as measured by VO_2max_ and MRS compared with the sedentary group. Considering that VO_2max_ improves as a result of increasing the workload intensity required to achieve physical conditioning (Wisløff et al., 2001), our data suggest that the training intensity adjustments was well conducted.

The latency, it means period between exposure to a cancer-causing agent and the growth of cancer, can provide important information regarding virulence of the tumor or to a decreased resistance of the host to the tumor (Westerlind et al., 2003; Ward et al., 2014). Latency was increased with the treadmill running training (50–70% of VO_2max_, 23–25 m/min, 30 min/day, 5 days/week, 15% grade), in the research of Westerlind et al. (2003). In the present study, during the second experimental phase, we observed that tumor latency was prolongate for all inoculated animals. When we recorded BW and food intake every 2 days, touching the inoculated paws. Interestingly, in some animals, evident tumor growth was manifested only on the fifth day after inoculation. It also may be related to the absence of the deaths in those rats, at least.

We received male rats at 21 days old from UEM *vivarium*. They were manipulated for five uninterrupted days for the treadmill running adaptation. In attempt to understand the delay tumor progression presented, we found behavioral studies that revealed resistance to disease and mortality factors in Walker 256 tumor-bearing rats (Ader and Friedman, 1965; Newton and Galvão, 2008). Rats separated from their mothers at 15 days were more susceptible to the transplanted carcinosarcoma than control animals weaned at 21 days (Ader and Friedman, 1965). In another investigation, the daily handling introduced in the immediate post-weaning period, similarly to what occurred in our study during exercise adaptation, was able to promote less emotional responsiveness and increased survival in inoculated rats at 45 days of age. We cannot disregard the control of cancer cell tumorigenicity studies, mainly in relation to genetic control of cancer growth *in vivo* (Wu and Pandolfi, 2001; Lewis et al., 2013).

Consistent with previous findings (Applegate et al., 1982; Calegari et al., 2011; Lira et al., 2011), TC rats had a lower BW and no reduction in food intake compared to SCs before tumor cell inoculation. The low BW was sustained in trained animals 15 days after inoculation. On the other hand, Walker 256 tumor-bearing sedentary rats did not lose overall weight over the course of the second experimental phase, analyzed by absolute values and weight of the carcass. In addition, all inoculated rats had lower food intake. It is recognized that this parameter reduces below need during tumor growth, contributing to the host depletion in cancer cachexia (Morrison, 1973; Lira et al., 2011; Cassolla et al., 2012; Aversa et al., 2017). However, Walker 256 tumor-bearing trained rats showed a reduction in anorexia compared to sedentary condition and did not exhibit apathy and lethargy that could characterize an advanced state of functional capacity loss.

Because the weight loss does not necessarily reflect the severity of cachexia in studies of cancer during tumor progression stages (Ebadi and Mazurak, 2014; Engelen et al., 2016), the cachexia index or loss of body mass after tumor removal has been used for this purpose (Cassolla et al., 2012; Trombini et al., 2015). Our study showed that Walker 256 tumor-bearing trained rats presented a reduction in this index compared their untrained counterparts. The reduction of anorexia also presented by these animals could explain the reduction of cachexia. Moreover, current studies suggest that cancer patients have an anabolic potential and prior to reaching the refractory phase of cachexia; exercise and good nutritional conditions could partially reverse this process (Lira et al., 2014; Engelen et al., 2016; Aversa et al., 2017).

Inflammatory conditions related to cancer cachexia (Gould et al., 2013; Lira et al., 2014) were indirectly evidenced in this research by the reduction in mechanical sensitivity in Walker 256 tumor-bearing rats. However, we demonstrated similar baseline responses in the nociceptive test in all experimental groups, suggesting that aerobic training performed before tumor cell inoculation did not provoke injuries in rats, which could interfere with proinflammatory muscle-derived humoral factors. This result corroborates with experimental investigations which suggested that physical exercise may be an effective method of managing inflammatory and neuropathic pain conditions. Voluntary wheel running and extended swimming attenuate the development of and reverse nerve injury-induced mechanical hypersensitivity in rodents (Kuphal et al., 2007; Sheahan et al., 2015).

In the present study, we also showed that low glucose and insulin levels before tumor cell inoculation did not revert even 15 days after the last training session. It has been shown that exercise increases glucose uptake by up to 50-fold through the simultaneous stimulation of delivery, transport across the muscle membrane and intracellular flux through metabolic processes (Sylow et al., 2017). Chronic exercise also improves and/or maintains blood glucose control (Zhu et al., 2008; van Dijk et al., 2012) and reduces blood insulin levels during intraperitoneal and intravenous glucose tolerance tests (Galbo et al., 1981; Calegari et al., 2011). Lower plasma insulin levels also been related in researches conducted by Gomes et al. (2013) and Veloso et al. (2018).

Hyperglycemia and hyperinsulinemia secondary to insulin resistance have been associated with an increased risk of cancer (Thomas et al., 2016; Adraskela et al., 2017). In both experimental phase, sedentary rats, inoculated or not, presented unaltered insulin sensitivity and a normal glucose/insulin profile. Except for the lack of physical activity, they did not present any risk factor for the development of cancer and/or tumor cell proliferation, including aging, obesity, central body fat distribution, type 2 diabetes, hyperglycemia, metabolic syndrome, each other’s (Godsland, 2010; Dankner et al., 2012). Considering the limitation that tumor incidence increases during older age and the current model uses young animals, previous studies conducted in our laboratory demonstrated similar results in tumor bearing control rats; they did not present insulin resistance neither hyperglycemia (Franco et al., 2014; Trombini et al., 2015).

Although the sedentary rats did not exhibit peripheral resistance to insulin during the tumor growth period in the present study, which has been evidenced in similar cancer models in the literature (Asp et al., 2010; Miksza et al., 2013), we pointed out several aspects in the interpretation of results that should be considered, such as animal species, associated insults, inoculated tissue and volume, experimental time, cellular viability, exercise protocol, drug administration, among others. The highly variable biological due these environmental interactions (Weiss and Harlos, 1979) and the lack of pure strains of cells, has been demonstrated long time ago, as a severe handicap to the study of malignant growths, particularly in Walker 256 tumor experimental model (Earle, 1935; Moraes et al., 2000).

It is known that decreased insulin sensitivity in skeletal muscle prevents the uptake of insulin and subsequently amino acids, thereby suppressing protein synthesis (Gould et al., 2013). On the other hand, moderate exercise training provokes an enhanced insulin sensitivity independently of the changes in body composition (McTiernan, 2008; Menshikova et al., 2017), and a reduction in insulin secretion in response to glucose (Galbo et al., 1981; Koranyi et al., 1991; Calegari et al., 2011; Gomes et al., 2013; Veloso et al., 2018); it also prevents islet failure and maintains overall islet quality (Delghingaro-Augusto et al., 2012; Gomes et al., 2013; Veloso et al., 2018). Contrasting findings showed an increase in insulin secretion in the islets of trained rats (Almeida et al., 2012; Tsuchiya et al., 2013). Discrepancies among studies could be attributed to responses to acute exercise protocols.

In our study, we showed improvements in insulin sensitivity and reductions in insulin secretion. The benefit of aerobic physical exercise in improving glucose homeostasis may justify the reduction in insulin secretion as a favorable mechanism for pancreatic function (Grassiolli et al., 2013). It has been shown that benefits of exercise continue after cancer diagnosis (Brown et al., 2012; Thomas et al., 2016), could be partially reverse the muscle wasting process or cachexia by increasing energy and nutrient disposal to healthy cells. Considering that tumor cells demonstrate abnormal glucose uptake to survive and proliferate (McTiernan, 2008; Ramanujan, 2015) and that insulin is a growth factor that can induce tumor cell proliferation (McTiernan, 2008; Arcidiacono et al., 2012; Brown et al., 2012; Kang et al., 2016; Adraskela et al., 2017), these factors likely influence tumor growth and metastasis in Walker 256 tumor-bearing adult (90 days old) rats.

It is a common outcome in numerous studies, that exercise training enhances beta-cell survival; there is no consensus that this practice induced changes on beta-cell functions (Grassiolli et al., 2015). We showed, for the first time, that aerobic exercise training started in young life partially preserved islet morphology (islet number and area), reduced pancreatic islet insulin content and reduced also the glucose-induced insulin secretion from Walker 256 tumor-bearing adult (90 days old) rats. Unfortunately, adult rats that were trained at adolescence, exhibited reduction in the beta-cell mass. The appropriately interpretation of this results should be considered, at least, in three aspects:

1. First, preserving beta-cell mass and function is fundamental to avoid metabolic disorders (Veloso et al., 2018), and concerning only this factor, would put doubts about our training protocol.
2. Second, the impact of aerobic exercise training cessation on pancreas morphology remained relatively unexplored. Influences of detraining on insulin sensitivity after 5 weeks of voluntary training were studied by Nagasawa et al. (1990). The main effect was observed only in the last 2 days after training cessation.
3. Third, similar data were showed by Amaral et al. (2015). They found a reduction on beta-cell mass in trained rats with metabolic syndrome, especially those that performed running protocol. This study suggests that the experimental model with low-intensity exercise seems to be more recommended for preventing morphological and metabolic pancreatic disorders. The benefits observed on the endocrine pancreas from rats that walking compared to those that running are related to the anti-inflammatory walking effects (Amaral et al., 2015).

Our study revealed that Walker 256 tumor-bearing sedentary rats demonstrated reduced both, beta-cell mass and pancreatic islet insulin content but without changing insulin secretion in isolated pancreatic islets. In contrast to the training effect, the inoculation of tumor cells reduced the number and area of islets in the sedentary animals. There was a disconnect between islet insulin stores and beta-cell function without impairing basal glucose levels. Evident glucose-insulin homeostasis was achieved by compensating for the reduced pancreatic islet insulin content and insulin secretion in trained animals. Despite some experimental limitations of the present study, we suggest that the expression and function of some proteins should be investigated to elucidate the reasons behind why tumor cell inoculation provoked changes in pancreatic morphology in Walker 256 tumor-bearing sedentary rats without impairing insulin secretion.

## Conclusion

The combined results shown in the current study suggest that aerobic exercise training applied at adolescence can be considered as a prevention cornerstone due to the decreased tumor growth, metastatic foci and cancer-related cachexia in Walker 256 tumor-bearing adult (90 days old) rats. Improvements in insulin sensibility, lower glucose and insulin levels and/or reduced insulin secretion stimulated by glucose may be implicated in this attenuation. These findings support the rationale for new studies involving basic and clinical investigations with a scientific translation approach needed to fill the gaps related to human outcomes.

## Author Contributions

VM designed and performed the experiments, analyzed the data, and wrote the paper. CdSF performed the experiments, supervised the project, and wrote the paper. KP, TR, IM, CP, AP, AdM, CM, DA, FF, AM, LT, SdSS, LS, KM, PdS, FdFS, GB, and MR performed the experiments and reviewed the manuscript. RG, GF, KP-R, HdS, TP, EV, RM, and JdO reviewed the manuscript. LdCL designed the experiments and reviewed the manuscript. WR supervised the project and reviewed the manuscript. PdFM designed the experiments, supervised the project, and wrote the paper.

## Conflict of Interest Statement

The authors declare that the research was conducted in the absence of any commercial or financial relationships that could be construed as a potential conflict of interest.
